# Bacterial Diversity of Water and Sediment Samples from Gull Point State Park (West Okoboji, Iowa) Determined Using 16S rRNA Gene Amplicon Sequencing

**DOI:** 10.1128/MRA.00726-21

**Published:** 2021-08-19

**Authors:** John A. Kyndt

**Affiliations:** a College of Science and Technology, Bellevue University, Bellevue, Nebraska, USA; University of Southern California

## Abstract

Gull Point State Park is located on a peninsula on the west shore of West Okoboji Lake (Iowa, USA). It is the primary state park in the Iowa Great Lakes region. Sediment and water samples from three locations at the Gull Point pond were analyzed for their microbial composition.

## ANNOUNCEMENT

**FIG 1:**
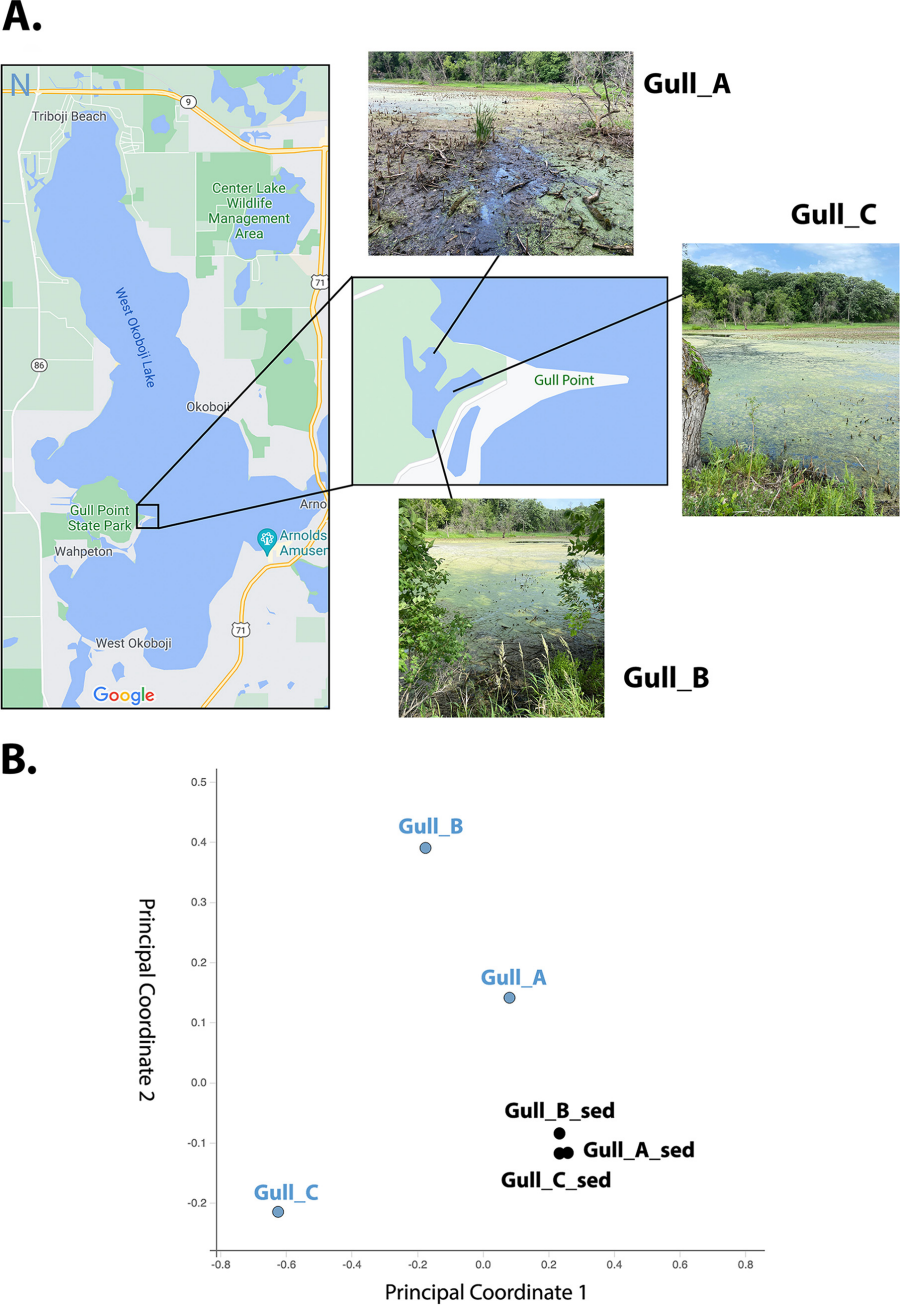
(A) Map of the sampling locations at Gull Point (West Okoboji Lake, IA, USA). Both the water and sediment samples were taken at three locations, Gull_A, Gull_B, and Gull_C. The maps were obtained from Google Maps. (B) Scatterplot of a principal coordinates analysis (PCoA) of the normalized relative abundance of all samples compared at the genus level. The water samples are shown in blue and the sediment samples in black.

The Gull Point and West Okoboji Lake landscape was created around 13,000 years ago during the last glacial period, when continental glaciers advanced and retreated (1, 2). Although the Gull Point State Park area is a popular vacation area, the pond that is located closest to West Okoboji Lake is part of a wildlife refuge and less accessed. No studies have been reported on the microbial diversity, and the pond area appears to be suffering from drought (in 2021) and the overgrowth of reed canary grass. To obtain a snapshot of the bacterial composition, samples were collected from three locations in July 2021 (Fig. 1A). At each location, we obtained a surface water sample and a sediment sample (where the pond was about 30 cm deep). The first sampling location was on the north side of the pond, closest to West Okoboji Lake (Gull_A/Gull_A_sed), while the second and third locations were on the east and south sides of the pond (Gull_B/Gull_B_sed and Gull_C/Gull_C_sed) (lat 43°22′16.69″N, long 95°9′41.62″W). Using sterile 15-ml collection tubes, 10-ml water samples and 5-ml sediment samples were collected, stored in a cooler with ice packs for ∼3 h, and transferred to the lab, where they were stored at 4°C for up to 2 days. Total DNA was extracted using the PureLink microbiome DNA purification kit (Invitrogen). Utilizing Qubit and NanoDrop, we determined the quality and quantity of the DNA, yielding *A*_260/280_ ratios between 1.60 (Gull_B) and 1.90 (Gull_A_sed). A 16S rRNA amplicon sequencing library was prepared for each sample, following the 16S metagenomic sequencing library preparation protocol (Illumina [3]). Amplicon primers targeting the V3 and V4 regions were synthesized using Sigma (4). The samples were sequenced using a 1.8 pM library with an Illumina MiniSeq sequencer. Paired-end (2 × 150-bp) sequencing generated the following numbers of reads: 373,010 (Gull_A), 452,056 (Gull_A_sed), 151,140 (Gull_B), 654,028 (Gull_B_sed), 371,190 (Gull_C), and 367,450 (Gull_C_sed). The primer sequences were removed, and reads with low-quality scores (average score, <20) were filtered out using the FASTQ Toolkit v2.2.0 within BaseSpace (Illumina). The 16S Metagenomics app v1.0.1 within BaseSpace was used to perform a taxonomic classification, with the Illumina-curated taxonomic database RefSeq RDP 16S v3 (5) and the RDP naïve Bayes taxonomic classification algorithm with an accuracy of >98.2% at the species level (6). Default parameters were used for all software unless otherwise specified.

In all samples, the most abundant phylum was *Proteobacteria* (31 to 66%), with smaller amounts of *Bacteroidetes* (3 to 15%), *Actinobacteria* (5 to 19%), and *Firmicutes* (6 to 11%). Over 72% of the reads were classified to the genus level. A principal coordinates analysis (PCoA) chart was generated within the 16S Metagenomics app, using Classical MDS on a Pearson covariance distance matrix generated from per-sample normalized classification abundance vectors (7, 8). Each sample vector was L1 normalized by multiplying every index by the inverse of the sum of the sample vector. The PCoA comparison of all samples at the genus level showed the three sediment samples to be more similar to each other than to the water samples at each location (Fig. 1B). In general, the sediment samples had a higher representation of *Draconibacterium*, *Thermomarinilinea*, and *Clostridium sensu stricto*, while the water phase samples showed more differentiation, with the following genera most represented: *Flavobacterium*, Gplla, and *Parcubacteria* (Gull_A); Mycobacterium and *Hydrogenophaga* (Gull_B); and *Malikia*, *Vogesella*, and *Hydrogenophaga* (Gull_C). These genera have been found in various environmental samples (9–18). Few to no typical coliform bacteria were identified, suggesting no significant contamination of the pond.

### Data availability.

The 16S rRNA gene amplicon data sets have been deposited at DDBJ/ENA/GenBank under BioProject accession number PRJNA746576 and can be accessed under SRA accession numbers SRR15140834 (Gull_A), SRR15141921 (Gull_A_sed), SRR15141697 (Gull_B), SRR15142125 (Gull_B_sed), SRR15142122 (Gull_C), and SRR15142121 (Gull_C_sed).
